# Items

**Published:** 1907-01

**Authors:** 


### ITEMS.

THE famous Bowery Mission Bread Line, now in its fourth year, at which every morning, at one o'clock, during the winter months, one thousand homeless and destitute men and boys are provided with a breakfast of hot coffee and rolls, resumed operations at Thanksgiving, midnight, and will continue to Easter morning, 1907. Last year, 144,000 were thus assisted, and altogether over half a million have had a weary night's tramp agreeably interrupted by this inexpensive, yet very welcome refreshment.

The Directors of the Bowery Mission have appointed Mr. John C. Earl, of 222 Bible House, New York City, Financial Secretary, succeeding Dr. Simon Trenwith, lately deceased.

---

"BEAUTY AS A FACTOR IN DISEASE."—The New York Pharmaceutical Company, Bedford Springs, Bedford, Mass., has just issued a most interesting and instructive booklet under the above title, which gives in detail the various methods adopted by the female sex of the many savage and semicivilised tribes to increase their attractiveness to the eyes of the male portion of their tribe or race. In some instances this so called improvement or attractiveness is carried to that degree of regional development that locomotion is impossible. A copy of the booklet will be sent upon application.

---

SPEAKING in reference to the wide use of effervescent beverages, it is admitted that carbonic acid gas in mineral waters greatly improves digestion, and that this is especially so in the case of Apollinaris, because its mineral constituents give additional help in that direction, so that Apollinaris is by far the best of the few naturally effervescent waters for mixing with whiskey, wine, fruit syrups, or milk. The consumption of effervescent liquids is especially large in Great Britain and the United States.

---

THE ANTIKAMNIA CHEMICAL COMPANY, as appears from their new advertisement in this issue, was prompt to file its guaranty under the new pure food and drugs act, their guaranty number being 10, showing that of all the food and drug manufacturers in the United States only nine filed their guaranty before that of this company. This action is but another instance of the promptitude of the Antikamnia Company to comply with the law in every particular.

---

THE MOET & CHANDON CHAMPAGNE, White Seal, vintage of 1900, is a superb wine for the sick. It should be prescribed by the physician and not taken by the patient at inappropriate times nor in improper quantities. It may, also, be stated with great propriety that it is the finest table champagne that is marketed at the present time. A trial of this celebrated wine for either purpose named will prove more convincing than mere words.

---

MESSRS. M. J. BREITENBACH COMPANY, 53 Warren Street, New York, are sending out their useful physician's memorandum book for 1907. It is one of the best desk diaries published and is so well known and appreciated that a description is unnecessary. A larger edition goes out this year than ever before but if any physician has been overlooked, an application made to the Breitenbach Company will secure the book by next post.

---

"THE ANTIPHLOGISTINE GIRL," a handsome photograph of a still more handsome nurse, in a Red Cross uniform, and in a tasteful frame, has been sent to the JOURNAL by the Denver Chemical Manufacturing Company, the makers of Antiphlogistine, for which we extend our best thanks.

